# Family medicine residency students’ competence in treatment planning for core diseases in the national core curriculum

**DOI:** 10.1017/S1463423626101613

**Published:** 2026-08-11

**Authors:** Berna Terzioğlu Bebitoğlu, Esra Akdeniz, Mehmet Ali Gülpınar

**Affiliations:** Eastern Mediterranean University: Dogu Akdeniz Universitesi, Cyprus

**Keywords:** core curriculum, medical education, prescribing, therapeutics

## Abstract

**Purpose:**

It was aimed to determine the competencies of Family Medicine residency students regarding the treatment planning of diseases coded with diagnosis-treatment in the ‘National Core Education Programme’ (NCC) in Turkey. We aimed to identify areas where residents feel less competent, which can inform curriculum improvements to better address gaps in treatment planning skills for core diseases.

**Methods:**

The study was conducted with Family Medicine residency students from one medical school selected from each geographical region of Turkey. Students were asked to assess themselves at the time of graduation from medical school and currently during their residency training, in terms of ‘appropriate drug selection’, ‘prescribing’, ‘non-drug treatment planning’ and ‘processing treatment management and giving information’ competencies for 59 diseases in the NCC.

**Results:**

The rate of Family Medicine residency students who felt competent in treatment planning/management when they graduated from medical school was very low; although this rate increased to a certain extent with residency training, it could not reach an acceptable rate. Despite partial improvements after graduation, the results suggest potential gaps in achieving the desired competency levels, even after 1–3 year of residency training. The residency students’ competency perceptions ranged from 10–30% to 60–80% across different diseases.

**Conclusion:**

It is important to review and reconstruct the six-year medical education and Family Medicine residency training programs, based on the framework drawn by NCC, focusing on rational treatment planning/management to reconceptualize treatment planning/management and decision-making.

## Introduction

Medical students’ competence in critical skills such as clinical reasoning and treatment planning is influenced by the curriculum, clinical experience, mentorship, and educational resources (Devine *et al*., 2020). Medical students’ perceived competence in treatment planning reveals a wide range of insights into how students develop confidence and competence in this essential aspect of clinical decision-making. Treatment planning is a critical skill that requires integrating knowledge of diseases, patient assessment, pharmacology, and evidence-based medicine to formulate appropriate therapeutic strategies. However, various studies have highlighted gaps in students’ competence in this area, influenced by the structure of medical education, clinical experience, and learning methods (General Medical Council, 2014; Ridley *et al*., 2024). Medical students often report feeling underprepared in pharmacology and the practical aspects of prescribing medications, which are central to treatment planning. The competence of medical students in treatment planning is a critical aspect of their education, impacting their future ability to provide safe and effective patient care.

General approach in medical education is based on educational outcomes and task-based learning (Harden *et al*., 2000). In our country, a similar approach has been underway since the 2000s. Nationwide, undergraduate and postgraduate education has been restructured and periodically updated within the framework of outcome- and task-based educational approaches. National Core Curriculum (NCC) for undergraduate education was developed in 2014 and updated in 2020 (Gülpınar *et al*., 2018; Council of Higher Education, 2020). It includes lists of problems and tasks (including the list of symptoms/conditions, the list of core diseases and clinical problems, and the list of basic medical practices).

The final year of the six-year undergraduate medical education program in our country is designated as the family medicine period. Additionally, a one-month family medicine internship program is defined for this period. In some faculties, apart from this, there are internship programs involving the Departments of Family Medicine during the first two years of clinical training (Durmaz and Turan, 2021). However, it is debatable to what extent the training programs in the final year of medical faculties are planned and implemented within the framework of primary care. Another topic of debate is how sufficient the three-year clinical training program is in addressing these needs. While studies discuss significant deficiencies in the competencies of medical school graduates, it is anticipated that these gaps can be addressed during the family medicine residency training process. However, there is no information in the literature regarding at what stage these deficiencies are addressed or whether they are truly resolved in practice. This study was conducted to explore answers to these questions, focusing specifically on treatment planning.

In our country, after graduation, medical doctors have the opportunity to enhance their primary care expertise by enrolling in a family medicine residency program during the postgraduate period. This program is designed to provide comprehensive training in the principles and practices of family medicine, equipping physicians with the skills necessary to deliver high-quality primary care services. Therefore, we wanted to reveal how family medicine residency students feel competent regarding treatment planning for core diseases. Evaluating how family medicine residency students feel about their competence in treatment planning can provide important insights into the effectiveness of the training program and identify areas for potential improvement in the curriculum. We also aimed to identify areas where residents feel less competent, which can inform curriculum improvements to better address gaps in treatment planning skills for core diseases. In this context, the research questions have been determined as follows:

**Main research question**

How competent do Family Medicine residency students perceive themselves in treatment planning for diseases coded with diagnosis and treatment in the National Core Education Program (2014 and 2020 versions)?

**Sub-questions:**

1. How competent do they feel in planning **medication therapy**?

- Selecting appropriate medications.
- Prescribing (including factors such as drug dosage, frequency of use, active ingredient quantity in pharmaceutical forms, route of administration, pharmaceutical form, adverse effects, potential interactions, treatment duration, and instructions).

1. How competent do they feel in planning and recommending **non-pharmacological treatments**?
2. How do Family Medicine residency students’ perceived competencies regarding medication and non-pharmacological treatment planning change during their **training years**?
3. At what specific stage of the training do students achieve the **target competency level** outlined in the National Core Education (NCC) Program regarding treatment planning?

## Methods

### Study population and sample

This quantitative study conducted with the participation of family medicine residency students from state university medical faculties selected from each region of Turkey, based on purposive sampling and inclusion/exclusion criteria. When selecting one medical faculty from each region, the inclusion criteria were state universities with at least 10 years of graduating medical students and an active family medicine residency program. The sole exclusion criterion was family medicine departments that had not admitted residency students in the past three years.

State universities were intentionally selected to ensure longer-established family medicine residency programs distributed across all geographical regions of Türkiye, allowing comparable representation while minimizing institutional variability. Consequently, although this sampling strategy strengthened internal consistency, the findings should be interpreted with caution when generalizing to private medical schools.

#### Participants

Ethical approval was obtained from ….. Clinical Research Ethics Committee with Decision No: KAEK 2021/KK /218-02.08.2021. After getting research ethics approval, one state medical faculty selected from each region according to criteria and the relevant departments were contacted for institutional permissions. Ultimately, the study was conducted at seven medical faculties that agreed to participate. The number of participants from each university is presented in Table 1.

**Table 1.** Number of residency students participating in the survey Table 1 long description.

Universities/region	Number of participants *n* (%)	Total number of learners (*n*)
Marmara University/Marmara region	27 (58.7)	46
Ondokuz Mayıs University/Black Sea region	30 (62.5)	48
Dicle University/Southeastern Anatolia region	4 (57.1)	7
Hacettepe University/Central Anatolia region	44 (97.8)	45
Atatürk University/Eastern Anatolia region	32 (80.0)	40
Çukurova University/Mediterranean region	17 (48.6)	35
Dokuz Eylül University/Aegean region	55 (68.8)	80
Total	209 (69.4)	301

### Data collection

To evaluate learners’ perceptions of competence regarding treatment planning within the scope of the research question, a questionnaire developed by the researchers was administered to family medicine residency students from the designated medical faculties. All students who were actively enrolled between 2021 and 2023 were included in the study. The residency students were asked to complete the questionnaire assessing their prior (thinking about the moment of graduation in the pre-graduation program at the graduation) and current level of confidence in the treatment management of core clinical problems/conditions listed in NCC of Turkey. The first section of the survey contains 11 questions inquiring about demographic information. In the second section, a total of 59 core diseases/clinical problems coded with diagnosis and treatment, as listed in the NCC 2014 and 2020, were selected (Appendix 1).

They were asked to rate each item on a 3-point Likert scale for level of competence, where 1 = not competent; 2 = partially competent and 3 = competent. For each item, participants were asked to rate their competence across different domains (pharmacological agent selection, dose, dosing frequency, prescribing, informing the patient/patient relatives, management of treatment with lifestyle counseling/non-drug treatment and referrals). The treatment plan for each listed core disease/clinical problem was evaluated separately in three stages: (1) appropriate drug selection and prescription, (3) non-drug treatment, and (4) providing information about the planned treatment.

### Data analysis

The categorical variables were presented by calculating their frequency distributions (number and percentage). When the knowledge level was taken as an ordinal response variable, a cumulative link mixed model (CLMM) was used to examine the changes over time, between groups, and across skills. Tukey correction was applied for post-hoc tests. The analyses were conducted using R version 4.4.0 (2024-04-24) with the lme4 and nlme packages. A *p*-value of <0.05 was considered statistically significant’.

## Results

### Participant characteristics

The mean age of the participants was 28.6 years (min: 24, max: 50), with a gender distribution of 34% male and 66% female. The participants had an average training duration of 16.1 months. There were 101 students in their first year of training, 48 in their second year, and 60 in their third year.

The findings related to Family Medicine residency students were analyzed across three groups. Since some participants started Family Medicine training immediately after graduation, while others worked in emergency healthcare services or primary care facilities for a period before beginning their training, the groups were formed as follows to evaluate the impact of their experience during this period:

Group 1: Residents who started Family Medicine immediately after graduation and are in their first year of training.

Group 2: Residents who worked for at least 6 months after graduation and are in their first year of Family Medicine training.

Group 3: Residents in their second year or beyond of Family Medicine training.

### Drug treatment planning

The findings were examined under three subheadings: ‘appropriate drug selection and prescribing, ‘planning non-drug treatment’, and ‘managing and informing about the treatment process’.

**Figure 1.**
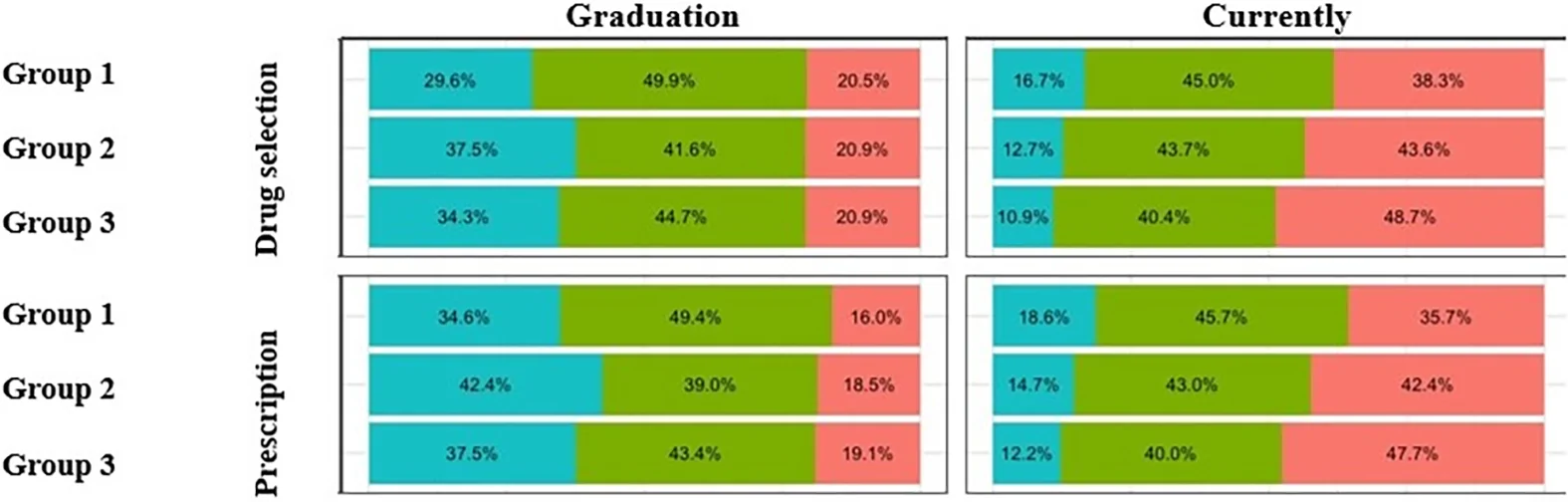
Family Medicine residents’ perceptions of appropriate drug selection and prescription competence (blue: not competent; green: partially competent; red: competent).

The responses provided by the groups regarding all diseases, based on the relevant competencies, are illustrated in Figure 1. The ratio of participants those who felt competent in appropriate drug selection and prescribing at graduation remained between 16.0% and 20.9%. With Family Medicine training, this rate only increased to 35.7% to 48.7%; however, it was found that a group of 10.9% to 18.6% still did not feel competent despite the time spent in residency training.

Subsequently, core diseases/clinical problems were evaluated by grouping them according to the organ systems defined in the NCC. The classification of the systems is as follows: sensory system diseases, respiratory system diseases, skin and soft tissue system diseases, circulatory/endocrine/hematopoietic system diseases, gastrointestinal system diseases, musculoskeletal system diseases, nervous behavior system diseases, urogenital system diseases, and multisystem diseases. Regarding the organ systems, the distribution of perceptions regarding appropriate drug selection and prescribing in the treatment plans are presented in Tables 2 and 3, respectively.

**Table 2.** Percentage distribution of learner competence perceptions regarding appropriate drug selection in treatment planning Table 2 long description.

Systems	Graduation (min–max)	Family medicine education	
		Group 1 and 2 (min–max)	Group 3 (min–max)
Sensory	7.7–32.4	17.8–77.8	28.8–72.1
Respiratory	5.6–36.5	12.5–80.0	12.7–82.4
Skin and soft tissue	11.5–32.7	18.8–65.2	36.5–64.1
Circulation, endocrine, and hematopoietic	9.6–42.3	17.4–78.7	24.0–88.5
Gastrointestinal	6.7–45.7	17.0–73.9	29.7–79.8
Musculoskeletal	6.1–35.2	10.9–68.9	19.4–69.6
Nervous system and behavior	11.5–26.5	17.8–45.7	26.2–42.2
Urogenital	2.0–37.1	8.3–68.1	33.0–69.3
Multisystem	6.1–50.0	10.6–85.4	16.5–85.3

**Table 3.** Percentage distribution of learner competence perceptions regarding appropriate prescription in treatment planning Table 3 long description.

Systems	Graduation (min–max)	Family medicine education	
		Group 1 and 2 (min–max)	Group 3 (min–max)
Sensory	10.2–30.5	17.8–75.6	27.9–72.1
Respiratory	4.2–30.5	14.6–82.2	12.6–81.4
Skin and soft tissue	6.1–29.0	22.9–60.9	36.9–62.1
Circulation, endocrine and hematopoietic	6.1–34.6	8.7–76.6	25.0–86.5
Gastrointestinal	6.1–43.8	19.1–73.9	32.7–76.9
Musculoskeletal	6.1–32.7	10.9–73.3	21.4–65.0
Nervous system and behavior	7.7–24.5	15.6–35.6	26.0–38.8
Urogenital	4.1–33.3	14.6–64.4	32.7–70.3
Multisystem	7.6–38.5	11.1–75.0	13.6–86.4

For drug selection and prescription of the diseases in sensory system, such as conjunctivitis, external otitis, otitis media, benign paroxysmal positional vertigo, and neonatal conjunctivitis, it was determined that the increase in the proportions of students feeling competent in their current assessments compared to graduation was statistically significant across all three groups (*p* < 0.05).

For drug selection and prescription in asthma, allergic rhinitis, and pneumonia, the increase in the proportions of students feeling competent in their current assessments compared to graduation was found to be statistically significant across all three groups (*p* < 0.05). In Group 2, there was an increase in the proportion of students feeling competent regarding pulmonary tuberculosis compared to graduation, while the proportion feeling competent regarding whooping cough significantly increased in Groups 2 and 3 (*p* < 0.05).

The ratio of students feeling competent in their current assessments compared to graduation was found to be statistically significant across all three groups (*p* < 0.05) for skin and soft tissue system diseases, circulation, endocrine, and hematopoietic system, gastrointestinal system and musculoskeletal system and nervous system. For conditions in urogenital system, such as sexually transmitted infections, genital infections, and premenstrual syndrome, the increase in the proportions of students feeling competent in their current assessments compared to graduation was found to be statistically significant across all three groups (*p* < 0.05). For urinary tract infections and hyperemesis gravidarum, the increase in the proportions of students feeling competent in their current assessments compared to graduation in the second and third groups was also found to be statistically significant (*p* < 0.05).

For infectious diseases with rashes, influenza, allergic reactions, urticaria, angioedema, diabetes mellitus and its complications, dyslipidemia, malnutrition, vitamin D deficiency, dehydration, megaloblastic anemia, drug side effects, and motion sickness, the increase in the proportions of students feeling competent in their current assessments compared to graduation was found to be statistically significant across all three groups (*p* < 0.05).

In brucellosis, the increase in the proportions of students feeling competent in drug selection in the first and second groups and in prescribing in the second and third groups compared to graduation was also found to be statistically significant (*p* < 0.05). For mumps and vitamin K deficiency, the increase in the proportions of students feeling competent in their current assessments compared to graduation in the second and third groups was statistically significant (*p* < 0.05). In diphtheria, there was a significant increase in the proportions of students feeling competent in drug selection only in the third group compared to graduation, while significant increases were noted in prescribing in the first and third groups (*p* < 0.05).

### Non-drug treatment planning

**Figure 2.**
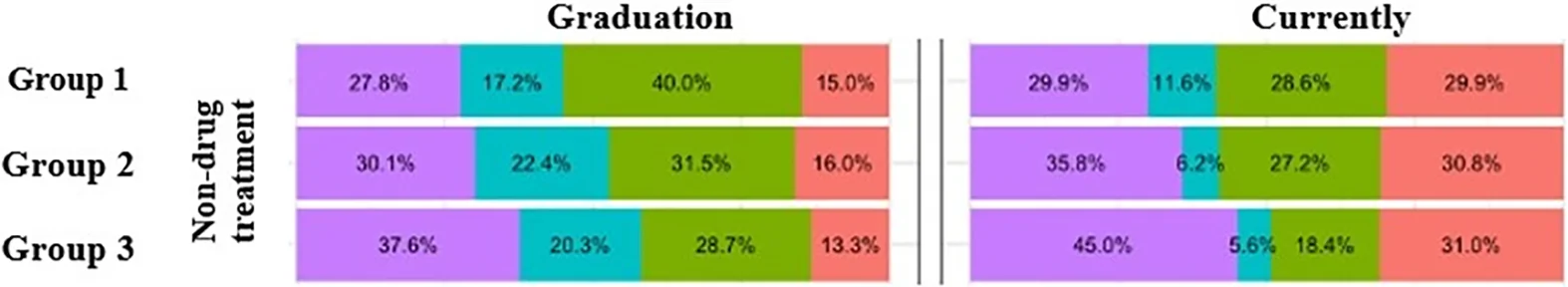
Family Medicine residents’ perceptions of competence regarding non-drug treatment planning (purple: no non-drug treatment; blue: not competent; green: partially competent; red: competent).

When all core diseases/clinical conditions are considered together, the percentage distributions regarding the perception of competence in non-drug treatment planning are presented in Figure 2.

The rate of residency students who said that there was no non-drug treatment at the time of graduation varied between 27.8% and 37.6% and currently between 29.9% and 45.0% (Table 4). The rate of students who said that there was a non-drug treatment and felt competent in this regard varied between 13.3% and 15.0% at graduation, while this rate increased and is now between 29.9% and 31.0% (Table 5). It was determined that residency students in all groups felt more competent in their current evaluations compared to when they graduated (*p* < 0.05).

**Table 4.** Percentage distribution of learner who thought that there was no non-drug treatment Table 4 long description.

Systems	Graduation (min–max)	Family medicine education	
		Group 1 and 2 (min–max)	Group 3 (min–max)
Sensory	48.9–78.6	57.1–75.7	41.2–54.9
Respiratory	34.7–85.4	40.5–83.3	27.1–69.4
Skin and soft tissue	34.7–83.3	60.5–80.6	50.7–70.1
Circulation, endocrine and hematopoietic	42.1–86.0	45.2–85.0	33.7–77.8
Gastrointestinal	56.8–90.9	60.0–89.5	47.2–80.6
Musculoskeletal	53.3–92.5	52.4–91.4	44.7–73.0
Nervous system and behavior	52.7–79.5	52.6–78.0	45.6–61.8
Urogenital	56.2–81.0	63.4–78.8	49.4–67.2
Multisystem	38.9–86.0	44.2–82.4	32.1–75.8

**Table 5.** Percentage distribution of learners who feel competent among those who thought that there is non-drug treatment Table 5 long description.

Systems	Graduation (min–max)	Family medicine education	
		Group 1 and 2 (min–max)	Group 3 (min–max)
Sensory	10.3–34.8	17.9–60.7	36.5–59.2
Respiratory	0–45.9	17.4–76.5	16.3–81.7
Skin and soft tissue	10.0–30.6	29.4–67.7	40.4–55.4
Circulation, endocrine and hematopoietic	7.4–37.8	26.1–66.7	17.1–83.3
Gastrointestinal	10.7–45.5	27.3–76.9	37.9–80.8
Musculoskeletal	6.2–34.3	22.2–34.3	28.8–64.8
Nervous system and behavior	15.2–25.7	28.6–50.0	41.5–53.8
Urogenital	9.3–35.3	17.6–56.7	36.2–67.7
Multisystem	4.0–37.0	21.4–68.4	21.1–76.3

These findings highlight variations in perceived competency across disease categories, with noticeable improvements during specialty training.

### Managing and informing about treatment

**Figure 3.**
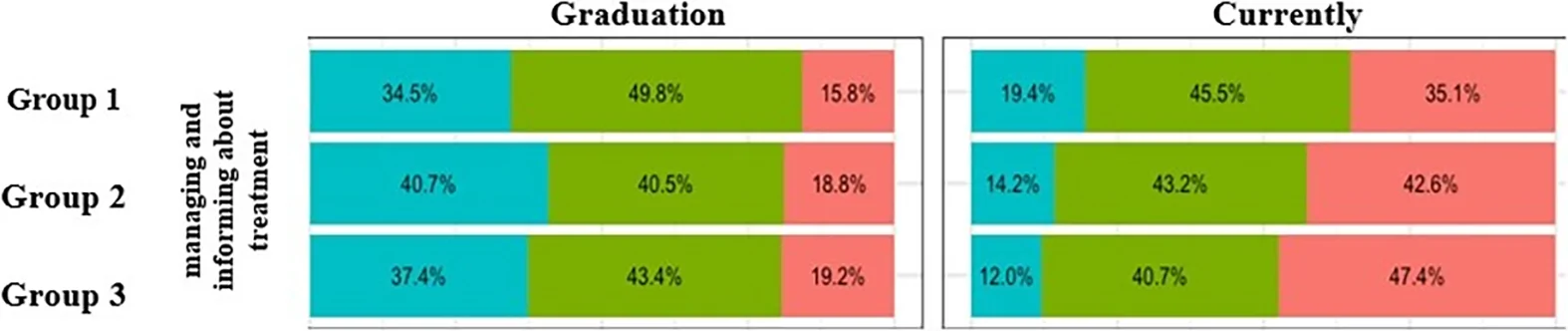
The perceptions of competence of Family Medicine residency students regarding managing and informing about treatment (blue: not competent; green: partially competent; red: competent).

The percentage distributions related to the perception of competence in managing and informing about treatment for all core diseases/clinical conditions are presented in Figure 3.

The proportion of residency students who feel competent in managing and informing about treatment at the time of graduation ranged between 15.8% and 19.2% (Table 6). This percentage has increased with residency training (currently) to 35.1–47.4%. When all diseases/clinical conditions are considered together, it was determined that in their current evaluations, participants in all groups felt more competent in managing treatment parameters compared to when they graduated (*p* < 0.05), although the percentages reached are still at a low level.

**Table 6.** Percentage distribution of learner competence perceptions regarding managing and informing about treatment Table 6 long description.

Systems	Graduation (min–max)	Family medicine education	
		Group 1 and 2 (min–max)	Group 3 (min–max)
Sensory	11.5–31.7	11.4–70.5	28.8–72.1
Respiratory	3.8–51.9	12.5–80.0	12.6–82.4
Skin and soft tissue	6.1–30.8	16.7–65.2	35.0–63.1
Circulation, endocrine and hematopoietic	5.8–40.4	15.2–76.6	23.1–85.6
Gastrointestinal	5.8–43.8	19.6–73.3	29.1–74.0
Musculoskeletal	6.1–34.6	15.2–68.9	20.4–65.0
Nervous system and behavior	9.6–24.5	9.6–24.5	26.9–36.5
Urogenital	2.0–34.3	12.5–65.9	29.8–69.3
Multisystem	6.1–36.5	10.6–70.8	18.6–85.4

### Evaluation of the most common diseases in Turkey

**Figure 4.**
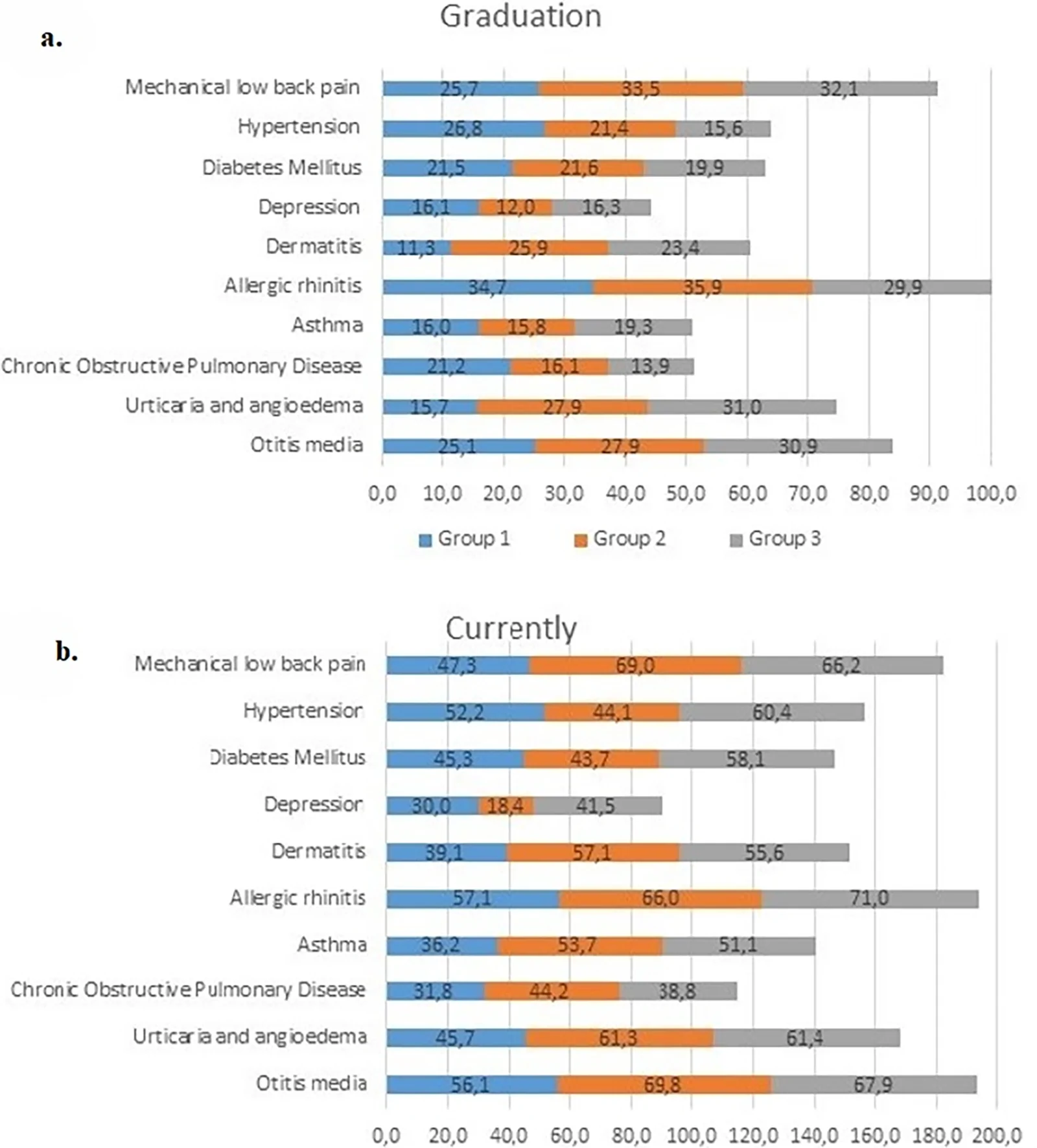
The ratios of residency students who feel competent regarding treatment planning for commonly seen diseases at the time of graduation (a) and currently (b).

According to the Turkey Health Survey Health Statistics-2022 data, the ranking of major diseases/health issues experienced by individuals aged 15 and older in our country is as follows: problems in the back and neck regions, hypertension, diabetes, allergies (allergic rhinitis, dermatitis), high blood lipids, asthma (including allergic asthma), arthrosis, depression, chronic obstructive pulmonary disease, coronary heart disease, Alzheimer’s disease (for those aged 65 and older), urinary incontinence, kidney problems, myocardial infarction, liver cirrhosis, failure, and stroke. The perceptions of competence of residency students regarding these commonly seen diseases at graduation and currently are presented in Figure 4a and 4b.

Among three groups, the percentage of learners who felt competent at graduation for mechanical low back pain was similar (25.7–33.5%), while these rates increased to 47.3% in Group 1, 69.0% in Group 2, and 66.2% in Group 3. For hypertension, the percentage of students who felt competent at graduation ranged between 15.6% and 26.8%, but this rate increased in all three groups to 52.2% in Group 1, 44.1% in Group 2, and 60.4% in Group 3. In diabetes mellitus, the percentages of students who felt competent at graduation were also close (19.9–21.5%), rising to 45.3% in Group 1, 43.7% in Group 2, and 58.1% in Group 3. For depression, the rates of feeling competent at graduation were similar (12.0–16.3%) but increased to 30% in Group 1, 18.4% in Group 2, and 41.5% in Group 3. In dermatitis treatment, the rates of feeling competent at graduation were 11.3%, 25.9%, and 23.4% for Groups 1, 2, and 3, respectively, while these rates increased to 39.1%, 57.1%, and 55.6% currently. In allergic rhinitis treatment planning, the rates of feeling competent at graduation were similar (29.9–35.9%) and increased to 57.1% in Group 1, 66.0% in Group 2, and 71.1% in Group 3. For asthma treatment planning, the rates of feeling competent at graduation were also close (15.8–19.3%), increasing to 36.2% in Group 1, 53.7% in Group 2, and 51.1% in Group 3. In chronic obstructive pulmonary disease, the rates of feeling competent at graduation were similar (19.9–21.5%), rising to 45.3% in Group 1, 43.7% in Group 2, and 58.1% in Group 3. For urticaria and angioedema, the rates of feeling competent at graduation were 15.7%, 27.9%, and 31.0% in Group 1 and have increased to 45.7%, 61.3%, and 61.4% in Groups 1, 2, and 3, respectively. Lastly, in otitis media, the rates of feeling competent at graduation were similar (25.1–30.9%) but increased to 56.1% in Group 1, 69.8% in Group 2, and 67.9% in Group 3. These findings indicate that despite residency training, the perceptions of competence have not fully reached the targeted level.

## Discussion

This is the first report on perceptions of family medicine residency students about their competency in treatment planning of all core diseases/clinical conditions listed in NCC in Turkey.

Despite partial improvements after graduation, the results suggest potential gaps in achieving the desired competency levels, even after 1–3 year of residency training. The residency students’ competency perceptions ranged from 10–30% to 60–80% across different diseases. This variation indicates challenges in achieving a standard baseline both among students and across medical schools. The competency perceptions of physicians at graduation were low. While these perceptions improved somewhat during family medicine residency training, they still did not reach an acceptable level. Across all conditions, perceived competency rates improved in all three groups post-graduation. This highlights the impact of residency training and practice experience. A systematic literature review on the prescribing competencies of final-year medical students revealed that many students lack the necessary preparedness, self-confidence, knowledge, and skills to prescribe safely and effectively. The review highlighted the need for a consensus on the essential prescribing competencies and called for significant changes in undergraduate clinical pharmacology and therapeutics education to improve future doctors’ prescribing abilities (Brinkman *et al*., 2018).

There are various studies showing that during the six-year medical education period, the competencies targeted by national frameworks, including those related to treatment, are often not achieved at the desired level. Similar to our findings, students have expressed concerns about their basic medical competencies and practices upon graduation, indicating that they feel insufficiently prepared for the profession (Watmough *et al*., 2009; Ross and Maxwell, 2012). In a study on prescribing ability of intern doctors in South Korea, it was reported that majority of the intern doctors felt that their undergraduate education was insufficient for safe- (75.8%) and evidence-based (68.9%) drug prescription (Gu *et al*., 2018). Thus majority of the participants in our study expressed that at the time of graduation they felt incompetent for most of the diseases in each system.

Considering the literature on rational drug use and rational treatment planning/management, it is evident that the integration of all sub-competencies is essential. However, students or new graduates may feel more or less competent in specific areas. For example, while most students can recall the names of drugs, they often struggle with determining dosages (Leape *et al*., 1991; Barber and Dean, 1998; Ross and Maxwell, 2012). Similarly, there can be wide variations in perceived competence levels depending on the disease.

In our study, no significant differences were observed in competency levels between those who began their residency training immediately after graduation and those who worked elsewhere for at least six months before starting training. Similarly, there were no meaningful differences among residency students in their second and third years of family medicine residency training for most diseases. These findings suggest that while family medicine residency training offers some improvement, its impact is insufficient. Furthermore, the consistently low competency levels at graduation across nearly all systems and groups indicate that six years of medical education is inadequate for planning the treatment of core diseases effectively.

Research focusing on primary care diseases, which are commonly managed in family medicine practice, has reported similar results. For instance, in a study examining the effectiveness of dermatology education during family medicine residency training, 72% of participants stated they struggled with diagnosing skin diseases, and 70.3% found treatment challenging. While 69.2% reported being capable of diagnosing and treating acne vulgaris, only 52.6% felt confident in the differential diagnosis and treatment of oral ulcers (Sönmez, 2021).

In this study, the highest perceived competency levels among residents for dermatological and soft tissue diseases were reported as follows: 65.2% for drug selection, 62.1% for prescription writing, 72.1% for management and patient counseling, and 67.7% for non-pharmacological recommendations. In another study involving primary care physicians, fewer than two-thirds of participants reported confidence in diagnosing (61.8%) and managing (58.0%) common dermatological conditions such as eczema, allergies, drug eruptions, and cutaneous infections (Lam *et al*., 2011).

In our study, prescription-writing skills were not assessed through practical application; instead, competency perceptions regarding elements of prescription were evaluated. These elements included appropriate drug selection, prescription-writing skills, dose determination, frequency of use, the active ingredient in pharmaceutical forms, dose amount, and the route of administration. The highest competency levels for drug selection and prescription writing were observed during residency training, with rates of 88.5% for selecting appropriate medication and 86.5% for prescribing in iron deficiency anemia. Similarly, in vitamin D deficiency, competency rates were 85.3% for drug selection and 86.4% for prescription writing. For common diseases like essential hypertension, the rates reached a maximum of 60.6% for drug selection and 56.7% for prescribing, while for diabetes mellitus, these rates were 59.6% and 57.3%, respectively. Overall, the competency rates for most systemic diseases were approximately one-third or less at graduation but increased to between one-half and two-thirds during residency training.

In a study evaluating prescription-writing competencies, the self-efficacy levels of general practitioners regarding ‘practical skills’ from the NCC were assessed. The competency levels for ‘writing accurate, appropriate, and legible prescriptions’ were reported as follows: 24.9% master level, 44.2% highly competent, 24.7% moderately competent, 3.6% minimally competent, and 1.6% completely incompetent (Ceylan, 2009). Another study involving primary care physicians from various regions of Turkey assessed their competency perceptions regarding the skills outlined in the NCC. Prescription writing was among the highest-rated skills, with 83% of participants feeling competent. However, this study evaluated overall perceptions rather than disease-specific competencies as in our study (Coşkun *et al*., 2021).

A study in Ireland involving graduates from five different medical faculties found that 91% of new graduates felt unprepared for all required skills/competencies during their internship. For prescription-writing competency, the rate of ‘highly competent’ was 7% before graduation and 32% during the internship. The rate of ‘competent’ was 47% before graduation and 54% during the internship, while the rate of ‘incompetent’ was 46% and 14%, respectively (Hannon, 2000).

Lifestyle modifications are a critical component of non-drug treatment planning. It is well-established that medication alone is often insufficient for managing many diseases, and lifestyle changes must accompany pharmacological interventions. For instance, in the treatment planning of essential hypertension, recommendations such as weight loss, regular aerobic exercise, salt restriction, and dietary modifications are highly significant. Failure to adhere to these changes may prevent the prescribed medication from achieving its expected efficacy. Guidelines on the non-pharmacological treatment of hypertension emphasize these recommendations (Mancia *et al*., 2023). In addition to chronic diseases, non-drug suggestions, such as adequate fluid intake, exercise, and maintaining body temperature, are also provided for some acute conditions. This study investigated students’ perceptions of competency regarding non-drug treatment planning alongside other sub-competencies. A significant improvement was observed in their perceived competency levels in non-drug treatments during their residency compared to their graduation.

For diseases like essential hypertension, asthma, and chronic obstructive pulmonary disease (COPD), where non-drug treatments are indispensable according to guidelines, some students mistakenly believe that these treatments are not applicable. For example, among second- and third-year residents, 28.6% believed there was no non-pharmacological treatment for COPD, while 45.0% of those who acknowledged its existence felt competent in planning such treatments. COPD guidelines emphasize non-pharmacological strategies, such as smoking cessation and avoiding harmful environmental exposures (Singh *et al*., 2019). Yet, in this study, less than half of the participants recognized non-pharmacological treatments for COPD, with only one-third feeling partially or fully competent in this area.

In essential hypertension, the proportion of participants acknowledging non-drug treatments ranged from 75.0% to 77.8%, while the percentage of those feeling competent in this domain ranged from 62.2% to 67.1%. Another common clinical issue in Turkey is mechanical low back pain, for which treatment guidelines recommend measures such as exercise and precautions when lifting heavy objects (Qaseem *et al*., 2017). In this study, 71% to 91.4% of participants recognized the existence of non-drug treatments for mechanical low back pain, while the highest rate of perceived competency in planning such treatments was 64.8%. This indicates a gap in achieving adequate competency in incorporating non-drug recommendations for managing mechanical low back pain, a prevalent condition in the community. Similarly, in dermatitis treatment, guidelines emphasize non-pharmacological recommendations, such as avoiding provoking factors and maintaining dietary precautions (Wollenberg *et al*., 2022). In this study, the proportion of participants who believed in non-pharmacological treatments for dermatitis was 70.0% to 72.2% during the first year of residency, but this decreased to 65.2% in subsequent years. The percentage of those feeling competent in this domain did not exceed 61.1%.

Studies evaluating rational drug use often examine whether non-pharmacological recommendations are included in patient education. In a study conducted among primary care physicians in Ankara, 74.3% reported providing non-pharmacological advice, while 25.7% indicated they only ‘sometimes’ make such recommendations (Doğukan, 2008). These findings highlight the need for enhanced emphasis on non-pharmacological treatment strategies during medical training, particularly in chronic disease management, where these interventions are critical for comprehensive patient care.

Knowledge competence for medication and management of treatment processes are different areas of competence. Accordingly, a study investigating the relationship between factual drug knowledge and treatment appropriateness among final-year medical students found a weak positive correlation between the two. This suggests that while factual drug knowledge is necessary, it is not sufficient for making appropriate treatment choices. The study emphasizes the need for training in therapeutic reasoning and decision-making including prescription writing, rather than solely focusing on factual knowledge (Brinkman *et al*., 2017). Students’ factual drug knowledge has limited influence on the appropriateness of their treatment decisions. Poor relationship between drug knowledge and the quality of treatment choices among general practitioners and recent medical graduates were previously demonstrated (Grad *et al*., 1997; Harding *et al*., 2010). Making appropriate treatment decisions is a complex cognitive skill that requires higher-order thinking processes. To develop this skill, medical students need structured, step-by-step training, such as the WHO Guide to Good Prescribing, which explicitly teaches students to assess the suitability of their treatment decisions for individual patients (de Vries *et al*., 1994; Brinkman *et al*., 2021).

A study validating a competence-based 360-degree assessment for advanced undergraduate medical students demonstrated that such assessments could effectively measure students’ performance in clinical contexts. The assessment, which included consultation hours, patient management, and patient handover phases, proved to be a valid tool for evaluating the competencies required for beginning residents. This approach underscores the importance of performance-based assessments in ensuring that medical students are well-prepared for real-world clinical challenges (Prediger *et al*., 2020). In another study his study evaluating how different levels of realism in learning contexts affect medical students’ prescribing competencies, it was demonstrated that increasing the realism of the learning context, particularly by involving students in patient care, significantly enhances prescribing competencies. Integrating real-world responsibility for patient care into training can have a substantial impact on improving students’ rational prescribing skills (Tichelaar *et al*., 2015).

The core competencies that are essential for medical education are well-documented. However, there are potential challenges of assessing and developing these skills. It is important that rather than compartmentalizing competencies, it is more beneficial to adopt a holistic and integrated approach to conceptualize, facilitate, and assess these skills in the context of real-world complexities. In the study of Xiangyun Du *et al*. (2019), ten key profession-related competencies that should be incorporated into the early stages of medical education are reported. Six of these – collaboration, communication, problem-solving, integrity, responsibility, and respect – were unanimously agreed upon by all participants, aligning with global literature and graduate attributes. However, planning and time management, critical thinking, and reflectivity were identified as areas needing improvement. Divergence arose around empathy and medical ethics, with faculty expressing concerns over how to effectively teach and assess these skills. These findings call for reforms in medical curricula to integrate essential skills early in students’ careers.

Family physicians are often the first to detect symptoms of chronic diseases and are responsible for ongoing management. They play a major role in handling these diseases and risk factors. The bulk of hypertension, diabetes, and respiratory illness should be managed by family doctors, not by specialists. Symptoms and signs of cardiovascular disease and cancer, such as chest pain, loss of appetite, and weight loss, are often noted by family physicians first. The nature of family practice is such that counseling regarding healthy diet, physical activity, and substance abuse is an integral part of a family practitioner’s work. Keeping this in mind, the family physician should be a central stakeholder. However, it is also demonstrated in other studies that there is need for more comprehensive training that includes early detection, patient education, and coordination of care with specialists (Kalra, 2009).

In our study, group 2 consistently shows strong post-graduation gains, particularly in mechanical low back pain, allergic rhinitis, and dermatitis. Group 3 often reflects the highest or competitive post-residency competency rates, particularly for hypertension, diabetes mellitus, and depression. While there is marked progress, the overall percentages (30–70% range) imply a need for further educational and training strategies to enhance confidence and readiness in treatment planning for certain conditions.

Similarly, a study investigating the self-efficacy beliefs of newly graduated physicians, especially regarding their perceived competency in managing conditions with the highest DALYs in Turkey, less than 70% of physicians reported feeling competent in most of the evaluated areas, highlighting substantial gaps in professional self-efficacy. They reported 50.4% of competency in diagnosis of diabetes, 39.5% competency in treatment of lower respiratory diseases and 42.3% competency in preventive measures for ischemic heart disease. Physicians reported relatively higher self-efficacy in management of low back pain (72.9%), neck pain (65.5%) and post-traffic accident cases (46.5%, though still suboptimal) (Aker and Midik, 2020).

In addition, moderate gains post-graduation indicates that managing chronic conditions such as Hypertension, Diabetes Mellitus and COPD might need more targeted and integrated training approaches, considering the multifaceted nature of these diseases.

The relatively low post-graduation competency in treating depression – especially in Group 2 – suggests gaps in mental health education or clinical exposure. Tailored modules in psychiatry could help. Skin and Allergic Conditions show significant post-graduation gains, particularly in Group 2, but remain areas where competency development may benefit from more practical exposure. Also there revealed a need for enhanced focus during training programs on treatment planning for respiratory conditions.

A survey of U.S. physicians revealed that most felt their training in chronic illness care was inadequate across multiple competencies, including geriatric syndromes, chronic pain, and patient education. Family practitioners reported better training compared to other specialists but still found gaps in several areas (Darer *et al*., 2004).

Training in lifestyle medicine, which focuses on modifying behaviors such as diet and physical activity, has shown promise. A program in Israel found that training in lifestyle medicine significantly improved physicians’ confidence and ability to counsel patients on healthy behaviors (Polak *et al*., 2016). In our study, a significant proportion of diseases were thought not be treated by non-drug treatment or lifestyle modifications by all groups.

The findings of this study have several implications for future curriculum development and competency-based medical education. First, the consistently low competency perceptions across several common clinical conditions indicate that treatment planning should be integrated longitudinally throughout undergraduate and residency education rather than being confined to isolated pharmacology or clinical clerkships. Second, competency gaps identified in chronic diseases, mental health, respiratory diseases, and non-pharmacological management support the development of targeted educational interventions, including case-based learning, simulation-based prescribing activities, workplace-based learning, and structured feedback. Third, assessment strategies should move beyond knowledge-based examinations toward authentic competency assessments, such as Objective Structured Clinical Examinations (OSCEs), workplace-based assessments, Mini-CEX, Direct Observation of Procedural Skills (DOPS), prescribing assessments, and Entrustable Professional Activities (EPAs). Such approaches would better evaluate learners’ ability to integrate clinical reasoning, pharmacotherapy, patient counseling, and shared decision-making in real clinical settings. Finally, the disease-specific competency profile generated in this study may help curriculum committees prioritize educational resources, identify areas requiring additional clinical exposure, and support future revisions of the NCC for both undergraduate and family medicine residency education.

### Conclusion and recommendations

The study highlights the value of residency training but underscores the need for targeted improvements in specific domains to ensure residents meet the expectations of their roles more comprehensively. Innovative educational strategies that incorporate early clinical exposure, structured feedback, integrated pharmacology training, and simulated environments may help address gaps in student preparedness for treatment planning. As the medical education landscape continues to evolve, focusing on these factors could lead to better-prepared physicians and improved patient care. Some of the suggestions that can be put forward in this framework are listed below.

- Enhance curriculum design: Focus on bridging the gaps in conditions with lower competency gains, such as depression, asthma, and chronic conditions, through hands-on, case-based training during residency. Enhanced pharmacology education, including case-based learning that involves prescription writing and drug management, significantly improves students’ confidence in formulating treatment plans. Rational treatment planning/management and planning internships through five to six longitudinal tracks, including one specifically for rational treatment planning/management, will enhance the quality of graduates.
- Evaluate learning approaches: Conduct a deeper analysis of teaching methods and their alignment with real-world requirements to ensure competencies are being effectively translated into practice.
- Practice-oriented educational activities: Planning learning activities focused on rational treatment planning/management, such as applied modules, case discussions, and courses, and conducting research to evaluate the effectiveness of these activities are crucial. Such studies will contribute to improving learning and assessment methods and tools in the field and strengthen the evidence base.

## Supporting information

10.1017/S1463423626101613.sm001 Terzioğlu Bebitoğlu et al. supplementary material Terzioğlu Bebitoğlu et al. supplementary material

## Data Availability

The datasets generated and analyzed during the current study are available from the corresponding author upon reasonable request.

## Supplementary material

The supplementary material for this article can be found at https://doi.org/10.1017/S1463423626101613.

## Table 1. Long description

A table titled 'Number of residency students participating in the survey' presents data on the number of participants and total number of learners from various universities and regions. The table has three columns: Universities/region, Number of participants n (%), and Total number of learners (n). It contains eight rows, including a total row. The universities and regions listed are Marmara University/Marmara region, Ondokuz Mayıs University/Black Sea region, Dicle University/Southeastern Anatolia region, Hacettepe University/Central Anatolia region, Atatürk University/Eastern Anatolia region, Çukurova University/Mediterranean region, and Dokuz Eylül University/Aegean region. The table shows the number of participants and the total number of learners for each university and region. For example, Marmara University/Marmara region has 27 participants (58.7%) out of 46 learners, Ondokuz Mayıs University/Black Sea region has 30 participants (62.5%) out of 48 learners, and so on. The total number of participants across all regions is 209 (69.4%) out of 301 learners.

Navigate back to Table 1..

## Table 2. Long description

A table comparing the percentage distribution of learner competence perceptions regarding appropriate drug selection in treatment planning across different organ systems. The table has 9 rows and 4 columns. The columns are labeled Systems, Graduation (min-max), and Family medicine education with sub-columns Group 1 and 2 (min-max) and Group 3 (min-max). The row labels are Sensory, Respiratory, Skin and soft tissue, Circulation, endocrine, and hematopoietic, Gastrointestinal, Musculoskeletal, Nervous system and behavior, Urogenital, and Multisystem. Each row lists the minimum and maximum percentages for each category. Notable trends include varying ranges of percentages across different systems and groups, indicating differences in learner perceptions of competence in drug selection for treatment planning.

Navigate back to Table 2..

## Table 3. Long description

A table comparing the percentage distribution of learner competence perceptions regarding appropriate prescription in treatment planning across different organ systems. The table has 9 rows and 4 columns. The columns are labeled Systems, Graduation (min-max), and Family medicine education with sub-columns Group 1 and 2 (min-max) and Group 3 (min-max). The row labels are Sensory, Respiratory, Skin and soft tissue, Circulation, endocrine and hematopoietic, Gastrointestinal, Musculoskeletal, Nervous system and behavior, Urogenital, and Multisystem. Each row lists the minimum and maximum percentages for each category. Notable trends include varying ranges of percentages across different systems and groups, indicating differences in learner competence perceptions.

Navigate back to Table 3..

## Table 4. Long description

A table comparing the percentage distribution of learners who thought there was no non-drug treatment across various systems and family medicine education groups. The table has 10 rows and 4 columns. The columns are labeled 'Systems', 'Graduation (min-max)', 'Family medicine education Group 1 and 2 (min-max)', and 'Family medicine education Group 3 (min-max)'. The rows list different systems and their corresponding percentage ranges. Row 1: Sensory, 48.9-78.6, 57.1-75.7, 41.2-54.9. Row 2: Respiratory, 34.7-85.4, 40.5-83.3, 27.1-69.4. Row 3: Skin and soft tissue, 34.7-83.3, 60.5-80.6, 50.7-70.1. Row 4: Circulation, endocrine and hematopoietic, 42.1-86.0, 45.2-85.0, 33.7-77.8. Row 5: Gastrointestinal, 56.8-90.9, 60.0-89.5, 47.2-80.6. Row 6: Musculoskeletal, 53.3-92.5, 52.4-91.4, 44.7-73.0. Row 7: Nervous system and behavior, 52.7-79.5, 52.6-78.0, 45.6-61.8. Row 8: Urogenital, 56.2-81.0, 63.4-78.8, 49.4-67.2. Row 9: Multisystem, 38.9-86.0, 44.2-82.4, 32.1-75.8.

Navigate back to Table 4..

## Table 5. Long description

A table comparing the percentage distribution of learners who feel competent among those who thought that there is non-drug treatment across different systems. The table has 11 rows and 4 columns. The columns are labeled Systems, Graduation (min-max), and Family medicine education with Group 1 and 2 (min-max), Group 3 (min-max). The rows are labeled with different systems: Sensory, Respiratory, Skin and soft tissue, Circulation, endocrine and hematopoietic, Gastrointestinal, Musculoskeletal, Nervous system and behavior, Urogenital, and Multisystem. Each row lists the minimum and maximum percentages for graduation and family medicine education groups. For example, the Sensory system has graduation percentages ranging from 10.3 to 34.8, Group 1 and 2 percentages ranging from 17.9 to 60.7, and Group 3 percentages ranging from 36.5 to 59.2. The table provides a detailed comparison of competence feelings among learners across different systems and education groups.

Navigate back to Table 5..

## Table 6. Long description

A table comparing learner competence perceptions in managing and informing about treatment across different groups. The table has 11 rows and 4 columns. The columns are labeled 'Systems', 'Graduation (min-max)', 'Family medicine education Group 1 and 2 (min-max)', and 'Family medicine education Group 3 (min-max)'. The rows are labeled with different systems such as Sensory, Respiratory, Skin and soft tissue, Circulation, endocrine and hematopoietic, Gastrointestinal, Musculoskeletal, Nervous system and behavior, Urogenital, and Multisystem. Each row contains minimum and maximum values for each group. Row 1: Sensory, 11.5-31.7, 11.4-70.5, 28.8-72.1. Row 2: Respiratory, 3.8-51.9, 12.5-80.0, 12.6-82.4. Row 3: Skin and soft tissue, 6.1-30.8, 16.7-65.2, 35.0-63.1. Row 4: Circulation, endocrine and hematopoietic, 5.8-40.4, 15.2-76.6, 23.1-85.6. Row 5: Gastrointestinal, 5.8-43.8, 19.6-73.3, 29.1-74.0. Row 6: Musculoskeletal, 6.1-34.6, 15.2-68.9, 20.4-65.0. Row 7: Nervous system and behavior, 9.6-24.5, 9.6-24.5, 26.9-36.5. Row 8: Urogenital, 2.0-34.3, 12.5-65.9, 29.8-69.3. Row 9: Multisystem, 6.1-36.5, 10.6-70.8, 18.6-85.4.

Navigate back to Table 6..
