# Supplementary material for: Family medicine residency students’ competence in treatment planning for core diseases in the national core curriculum
Source: Prim Health Care Res Dev. 2026 Aug 11;27:e90. doi: 10.1017/S1463423626101613 (PMC13740163; doi:10.1017/S1463423626101613)
Supplement: Supplementary file 1 [file S1463423626101613sup001.docx]

**APPENDIX 1.**

**National Core Curriculum (NCC) Core Diseases/Clinical Problems**

**Sensory System Diseases**

- Conjunctivitis
- Otitis externa
- Otitis media
- Benign paroxysmal positional vertigo
- Neonatal conjunctivitis

**Respiratory System Diseases**

- Lung tuberculosis
- Asthma
- Allergic rhinitis
- Whooping cough
- Pneumonias
- Upper respiratory tract infections
- Chronic obstructive pulmonary disease (COPD)

**Skin and Soft Tissue System Diseases**

- Acne vulgaris/rosacea
- Pressure ulcers
- Parasitic diseases of the skin
- Skin and soft tissue infections, abscesses
- Dermatitis (atopic, contact, seborrheic, diaper)
- Burns

**Circulation, Endocrine, and Hematopoietic System Diseases**

- Essential hypertension
- Hypothyroidism
- Congenital hypothyroidism
- Iron deficiency anemia
- Goiter

**Gastrointestinal System Diseases**

- Peptic diseases
- Gastroesophageal reflux
- Gastroenteritis
- Gastrointestinal parasitic diseases
- Moniliasis
- Functional constipation in children
- Stomatitis
- Hemorrhoids
- Anal fissure

**Musculoskeletal System Diseases**

- Mechanical low back pain
- Osteoarthritis
- Tenosynovitis

**Nervous System and Behavior**

- Depression
- Febrile convulsion
- Tension-type headache

**Urogenital System Diseases**

- Sexually transmitted infections
- Genital infections
- Premenstrual syndrome
- Urinary system infections
- Hyperemesis gravidarum

**Multisystem Diseases**

- Exanthematous infectious diseases
- Influenza
- Allergic reactions
- Urticaria and angioedema
- Diabetes mellitus and complications
- Dyslipidemia
- Malnutrition
- Vitamin D deficiency
- Dehydration
- Megaloblastic anemia
- Drug side effects
- Motion sickness
- Brucellosis
- Mumps
- Vitamin K deficiency
- Diphtheria
